# The C-terminal Residues of *Saccharomyces cerevisiae* Mec1 Are Required for Its Localization, Stability, and Function

**DOI:** 10.1534/g3.113.006841

**Published:** 2013-10-01

**Authors:** Lance F. DaSilva, Samantha Pillon, Julie Genereaux, Megan J. Davey, Gregory B. Gloor, Jim Karagiannis, Christopher J. Brandl

**Affiliations:** * Department of Biochemistry, Schulich School of Medicine & Dentistry, The University of Western Ontario, London, Canada N6A5C1; † Department of Biology, The University of Western Ontario, London, Canada N6A5B7

**Keywords:** Mec1, FATC domain, PIKK proteins, Rpn3

## Abstract

Mec1, a member of the phosphoinositide three-kinase-related kinase (PIKK) family of proteins, is involved in the response to replicative stress and DNA damage and in telomere maintenance. An essential 30 to 35 residue, the FATC domain is found at the C-terminus of all PIKK family members. To investigate the roles of the C-terminal residues of Mec1, we characterized alleles of *Saccharomyces cerevisiae mec1* that alter the FATC domain. A change of the terminal tryptophan to alanine resulted in temperature-sensitive growth, sensitivity to hydroxyurea, and diminished kinase activity *in vitro*. Addition of a terminal glycine or deletion of one, two, or three residues resulted in loss of cell viability and kinase function. Each of these Mec1 derivatives was less stable than wild-type Mec1, eluted abnormally from a size exclusion column, and showed reduced nuclear localization. We identified *rpn3-L140P*, which encodes a component of the 19S proteasomal regulatory particle of the 26S proteasome, as a suppressor of the temperature-sensitive growth caused by *mec1-W2368A*. The *rpn3-L140P* allele acted in a partially dominant fashion. It was not able to suppress the inviability of the C-terminal truncations or additions or the hydroxyurea sensitivity of *mec1-W2368A*. The *rpn3-L140P* allele restored Mec1-W2368A to nearly wild-type protein levels at 37°, an effect partially mimicked by the proteasome inhibitor MG-132. Our study supports a role for the C-terminus in Mec1 folding and stability, and suggests a role for the proteasome in regulating Mec1 levels.

Members of the phosphoinositide three-kinase-related kinase (PIKK) family of proteins are important in the cellular response to various forms of stress (Abraham 2004). The PIKK proteins are large (for example, Tra1 and Mec1 are 3744 and 2368 residues, respectively) and are characterized by a C-terminally positioned domain that resembles the phosphatidylinositol-3 kinases (PI3K; Abraham 2004; Lempiäinen and Halazonetis 2009; Lovejoy and Cortez 2009).

Three members of the PIKK family have principal roles in DNA damage-response pathways. Ataxia telangiectasia–mutated (ATM; Tel1 in *Saccharomyces cerevisiae*), ataxia telangiectasia and Rad3-related (ATR; Mec1 in *S. cerevisiae*), and the DNA-dependent protein kinase catalytic subunit (DNA-PKc) transmit and amplify the damage signal through the phosphorylation of target proteins. Recruitment to sites of DNA damage is critical for their activation and function in checkpoint signaling and DNA repair (Falck *et al.* 2005). ATM/Tel1 acts principally in response to double-strand breaks, whereas ATR/Mec1 responds to a number of DNA insults, particularly replicative stress caused by stalled replication forks (Labib and De 2011). Both have roles in the maintenance of stable telomeres (Tomita and Cooper 2008; Moser *et al.* 2011; Yamazaki *et al.* 2012). DNA-PKc acts with Ku70 and Ku80 in nonhomologous end-joining of double-strand breaks; a direct homolog has not been found in *S. cerevisiae*.

Mec1/ATR is an essential gene (Cimprich and Cortez 2008). It is recruited to stalled replication forks and sites of DNA damage through the direct interaction of its associated protein Lcd1/Ddc2 with RPA-coated single-strand DNA (Rouse and Jackson 2002; Zou and Elledge 2003; Katou *et al.* 2003; Osborn and Elledge 2003; Ball *et al.* 2005). This recruitment is part of a series of events that results in the phosphorylation of regulatory and effector molecules that activate a cell-cycle checkpoint or apoptotic signals. Mec1 target molecules include histone H2A (Downs *et al.* 2000; Cobb *et al.* 2005), RPA (Brush *et al.* 1996), components of the Mcm2-7 DNA helicase complex (Cortez *et al.* 2004; Yoo *et al.* 2004), the Ino80 subunit Ies4 (Morrison *et al.* 2007), and the effector kinases Rad53 and Chk1 (Pellicioli *et al.* 1999; Sweeney *et al.* 2005; Ma *et al.* 2006). Fork stabilization is the essential function of Mec1 (Desany *et al.* 1998; Friedel *et al.* 2009) and occurs through multiple mechanisms, including retaining DNA polymerase at the replication fork (Cobb *et al.* 2003). In addition, Mec1 is required to activate the expression of ribonucleotide reductase, thus enhancing deoxyribonucleotide synthesis after DNA damage and perhaps during normal S phase (Zegerman and Diffley 2009). This function can be bypassed by increasing deoxyribonucleotide levels either by overexpressing Rnr3, one of the catalytic subunits of ribonucleotide reductase, or by deletion of the ribonucleotide reductase inhibitor Sml1 (Desany *et al.* 1998; Zhao *et al.* 1998). Interestingly, Mec1 and ATR are required to prevent chromosome breaks even in the absence of genotoxic stress. Augmenting deoxyribonucleotide levels does not rescue the checkpoint defects or DNA damage sensitivity of cells lacking functional Mec1 (Zhao *et al.* 1998).

Other PIKK family members include SMG-1 (suppressor with morphological effect on genitalia family member), target of rapamycin (TOR), and transformation/transcription domain-associated protein (TRRAP). SMG-1 is found in metazoans. It has many similar roles related to genotoxic stress (Brumbaugh *et al.* 2004; Gehen *et al.* 2008) and telomere stability (Azzalin *et al.* 2007) as ATM and ATR, and it is also the key signaling molecule required for the nonsense-mediated decay pathway (Chang *et al.* 2007). TOR is found in two complexes, TORC1 and TORC2. TORC1 integrates nutrient and growth factor signals, inducing anabolic pathways including protein synthesis and inhibiting catabolic pathways (Loewith and Hall 2011). TORC2 is involved in spatial control of cell growth by regulating the actin cytoskeleton (Cybulski and Hall 2009). The only member of the PIKK family that is not a Ser/Thr kinase is the transcriptional cofactor TRRAP (McMahon *et al.* 1998; Saleh *et al.* 1998). The *S. cerevisiae* homolog Tra1 is an essential component of the multisubunit, multifunctional SAGA and NuA4 histone acetyltransferase complexes (Grant *et al.* 1998; Saleh *et al.* 1998). A key role for Tra1 is to recruit HAT complexes to promoters via its association with transcriptional activators (Brown *et al.* 2001; Bhaumik *et al.* 2004; Fishburn *et al.* 2005; Reeves and Hahn 2005).

As well as having the PI3K domain, the PIKK proteins share a number of other features. N-terminal to the PI3K domain is a FAT (FRAP-ATM-TRRAP) domain that consists largely of helical HEAT (Huntington, elongation factor 3, PR65/A, TOR) and tetratricopeptide (TPR) repeats; in fact, these repeats extend through to the N-termini, making most of the protein helical (Bosotti *et al.* 2000; Perry and Kleckner 2003; Sibanda *et al.* 2010; Knutson and Hahn 2011). The C-terminal to the PI3K domain is a less highly conserved PIKK regulatory domain (PRD; Mordes *et al.* 2008). At the C-terminus of the PIKK molecules is the 30 to 35 residue FATC domain (FAT C-terminal; Bosotti *et al.* 2000). The structure of the isolated FATC domain of *S. cerevisiae* Tor1 (Dames *et al.* 2005) is helical, with a C-terminal loop held in place by a disulphide linkage. Although the cysteines are not conserved and the cellular FATC domain is not likely to always exist in an oxidized state, the helical structure is likely conserved (Lempiäinen and Halazonetis 2009; Sturgill and Hall 2009). The crystal structure of mammalian target of rapamycin (mTOR) including the FAT through to the C-terminus reveals that the FATC domain is an integral part of the kinase, positioned adjacent to the activation loop (Yang *et al.* 2013). The substrate-binding groove also includes portions of the FATC domain. The importance of the FATC domain has been demonstrated with molecular studies. Deletion of the C-terminus of mTOR or mutation of a conserved tryptophan five residues from the C-terminus eliminates kinase activity (Takahashi *et al.* 2000). Similar mutations within the FATC domains of DNA-PKcs and SMG-1 cause a loss of kinase activity (Priestley *et al.* 1998; Beamish *et al.* 2000; Morita *et al.* 2007). As suggested by Lempiäinen and Halazonetis (2009), the FATC domain likely regulates the kinase domain through interactions with the activation loop similar to the helical domains of the PI3 kinases. The FATC domain is also critical for the function of Tra1. Addition of as little as a single glycine to the C-terminus of Tra1 results in loss of cellular viability (Hoke *et al.* 2010). A strain with a mutation of the terminal phenylalanine residue of Tra1 to alanine (*tra1-F3744A*) shows growth defects, including temperature sensitivity and slow growth on media containing ethanol, Calcofluor white, or rapamycin. The F3744A mutation also results in mislocalization of the protein to the cytoplasm, particularly under conditions of stress (Genereaux *et al.* 2012). We identified a partially dominant mutation within *tti2* as a suppressor of *tra1-F3744A* (Genereaux *et al.* 2012). *TTI2* encodes a component of the Tel2-Tti1-Tti2 complex that associates with chaperone proteins in the folding of PIKK proteins (Takai *et al.*, 2007, 2010; Horejsí *et al.*, 2010; Hurov *et al.*, 2010; Kaizuka *et al.*, 2010). This suggests that the C-terminal residue of the molecule is required for folding and was consistent with the *tti2* suppressor mutation decreasing the proteolytic degradation of Tra1-F3744A and increasing its nuclear localization.

To determine if the C-terminus of the FATC domain is a general requirement for the folding and function of the PIKK proteins, we have examined alleles of *mec1* that disrupt this domain. Addition or deletion of residues results in loss of viability and kinase activity. Proteins containing these insertions or deletions, as well as a C-terminal change of tryptophan to alanine, have reduced nuclear localization and elute from a gel filtration column with an abnormally high molecular mass. They are also less stable after isolation than the wild-type protein. A second site mutation of *rpn3-L140P*, which encodes a component of the 19S proteasomal regulatory particle, restores the levels of the Mec1-W2368A protein and growth of the *mec1-W2368A* strain at 37°, further supporting a role for the C-terminus in protein folding and stability.

## Materials and Methods

### Yeast strains and growth

Yeast strains are listed in Table 1 and are derivatives of the diploid strain BY4743 (Winzeler and Davis 1997). Strains containing one Flag^5^-tagged (CY6172) or eGFP-tagged (CY6295) *MEC1* allele, marked with *URA3*, were made by one-step integration of the *Sph*I-*Eco*RI fragments of pCB2363 and pCB2395, respectively. Expression of these alleles is driven by the *TRA1* promoter. Integration of the *mec1-W2368A* allele has been described previously (Genereaux *et al.* 2012). This strain, strains with C-terminal truncations of one, two, or three residues (*mec1-Δ1*, *Δ2*, and *Δ3*), and the strain with the addition of a glycine residue (*mec1-2369G*) were similarly integrated as *Sph*I-*Sac*I fragments of the *HIS3*-tagged allele. Haploid strains containing *mec1-W2368A* (CY6175) or wild-type *MEC1* (CY6194) were generated by sporulation of their respective diploid strains. Haploid strains containing *mec1* deletion and addition alleles were made by integrating the *mec1* allele into CY6319 or CY6321 *MAT****a*** *sml1*::*KanMX* deletion strains derived from the consortium collection and containing *eGFP-MEC1 or Flag^5^-MEC1*, respectively. Diploid strains expressing eGFP-tagged Mec1, Mec1-W2388A, Mec1-Δ1, Mec1-Δ2, or Mec1-2369G, and RFP-tagged Nic96 were made by mating of CY6307, CY6302, CY6330, CY6344, and CY6342 with the RFP-tagged *NIC96* strain in the EY0987 background (*MAT***α** *his3Δ1 lys2Δ0 ura3Δ0*; Huh *et al.* 2003; kindly provided by Peter Arvidson). CY6265 containing *mec1-W2368A* and *rpn3-L140P* was isolated in the selection scheme described. CY6398 (*rpn3-L140P*) was obtained after backcrossing with BY4741. The diploid strain CY6400 (*MEC1/Flag^5^-mec1-W2368A rpn3-L140P/rpn3-L140P*) was obtained by mating of CY6391 and CY6399. CY6418 (*tra1-W3744A rpn3-L140P*) was obtained after crossing with CY4350 (Genereaux *et al.* 2012) and selecting for spore colonies that grew in the absence of histidine and carried the *rpn3-L140P* allele as determined by sequencing. CY6465 was derived from CY6076 by integrating *rpn3-L140P* contained in the *URA3*-containing yeast integrating plasmid YIPlac211 (CB2457) after digestion with *Bsa*B1.

**Table 1:** Strains used in this study

Strain	Genotype	Reference
BY4743	*MAT****a****/****α*** *his3Δ1/his3Δ1 leu2Δ0/leu2Δ0 LYS2/lys2Δ0 met15Δ0/MET15 ura3Δ0/ura3Δ0*	Winzeler and Davis (1997)
BY4741	*MAT***a** *ura3Δ0 met15Δ0 his3Δ0 leu2Δ0*	Winzeler and Davis (1997)
BY4742	*MAT***α** *ura3Δ0 lys2Δ0 his3Δ0 leu2Δ0*	Winzeler and Davis (1997)
CY4350	*MAT****a*** *ura3Δ0 his3Δ0 leu2Δ0 tra1-F3744A-HIS3*	Hoke *et al.* (2010)
CY4353	*MAT****a*** *ura3Δ0 his3Δ0 leu2Δ0 TRA1-HIS3*	Hoke *et al.* (2010)
CY6076	*MAT***a** *ura3Δ0 his3Δ0 leu2Δ0 mec1-W2368A-HIS3*	Genereaux *et al.* (2012)
CY6077	*MAT***α** *ura3Δ0 his3Δ0 leu2Δ0 mec1-W2368A-HIS3*	This work
CY6106	*MAT***a** *ura3Δ0 his3Δ0 leu2Δ0 mec1-W2368A-URA3*	This work
CY6172	Isogenic to BY4743 except *MEC1*/*URA3-Flag^5^-MEC1*	This work
CY6175	*MAT***a** *ura3Δ0 his3Δ0 leu2Δ0 URA3-Flag^5^-mec1-W2368A-HIS3*	This work
CY6192	Isogenic to BY4743 except *MEC1*/*URA3-Flag^5^-mec1-W2368A-HIS3*	This work
CY6184	Isogenic to BY4743 except *MEC1*/*URA3-Flag^5^-mec1-Δ1-HIS3*	This work
CY6194	*MAT***a** *ura3Δ0 his3Δ0 leu2Δ0 URA3-Flag^5^-MEC1*	This work
CY6203	Isogenic to BY4743 except *MEC1*/*URA3-Flag^5^-mec1-2369G-HIS3*	This work
CY6233	Isogenic to BY4743 except *MEC1*/*URA3-Flag^5^-mec1-Δ3-HIS3*	This work
CY6250	Isogenic to BY4743 except *MEC1*/*URA3-Flag^5^-mec1-Δ2-HIS3*	This work
CY6265	*MAT***α** *ura3Δ0 his3Δ0 leu2Δ0 mec1-W2368A-URA3 rpn3-L140P*	This work
CY6291	Isogenic to BY4743 except *LEU2-MEC1*/*URA3-eGFP-MEC1-Δ1-HIS3*	This work
CY6292	Isogenic to BY4743 except *LEU2-MEC1*/*URA3-eGFP-MEC1-Δ3-HIS3*	This work
CY6293	Isogenic to BY4743 except *LEU2-MEC1*/*URA3-eGFP-mec1-2369G-HIS3*	This work
CY6294	Isogenic to BY4743 except *LEU2-MEC1*/*URA3-eGFP-MEC1-Δ2-HIS3*	This work
CY6295	Isogenic to BY4743 except *MEC1*/*URA3-eGFP-MEC1*	This work
CY6296	Isogenic to BY4743 except *MEC1*/*URA3-eGFP-mec1-W2368A-HIS3*	This work
CY6302	*MAT***a** *ura3Δ0 his3Δ0 leu2Δ0 URA3-eGFP-mec1-W2368A-HIS3*	This work
CY6303	*MAT***α** *ura3Δ0 his3Δ0 leu2Δ0 URA3-eGFP-mec1-W2368A-HIS3*	This work
CY6306	*MAT***α** *ura3Δ0 his3Δ0 leu2Δ0 URA3-eGFP-MEC1*	This work
CY6307	*MAT***a** *ura3Δ0 his3Δ0 leu2Δ0 URA3-eGFP-MEC1*	This work
CY6319	*MAT***a** *ura3Δ0 his3Δ0 leu2Δ0 sml1Δ0*::*KanMX URA3-eGFP-MEC1*	This work
CY6321	*MAT***a** *ura3Δ0 his3Δ0 leu2Δ0 sml1Δ0*::*KanMX URA3- Flag^5^-MEC1*	This work
CY6330	*MAT***a** *ura3Δ0 his3Δ0 leu2Δ0 sml1Δ0*::*KanMX URA3-eGFP-mec1-Δ1-HIS3*	This work
CY6342	*MAT***a** *ura3Δ0 his3Δ0 leu2Δ0 sml1Δ0*::*KanMX URA3-eGFP-mec1-2369G-HIS3*	This work
CY6344	*MAT***a** *ura3Δ0 his3Δ0 leu2Δ0 sml1Δ0*::*KanMX URA3-eGFP-mec1-Δ2-HIS3*	This work
CY6349	*MAT****a*** *ura3Δ0 his3Δ0 leu2Δ0 sml1Δ0*::*KanMX URA3-Flag^5^-mec1-2369G-HIS3*	This work
CY6391	*MAT***α** *ura3Δ0 his3Δ0 leu2Δ0 mec1-W2368A-HIS3 rpn3-L140P*	This work
CY6398	*MAT***a** *ura3Δ0 his3Δ0 leu2Δ0 rpn3-L140P*	This work
CY6399	*MAT***α** *ura3Δ0 his3Δ0 leu2Δ0 rpn3-L140P*	This work
CY6400	*MAT***a/α** *ura3Δ0/ura3Δ0 his3Δ0/his3Δ0 leu2Δ0/leu2Δ0 URA3- Flag^5^-mec1-W2368A/MEC1 rpn3-L140P/rpn3-L140P*	This work
CY6418	*MAT****a*** *ura3Δ0 his3Δ0 leu2Δ0 tra1-F3744A-HIS3 rpn3-L140P*	This work
CY6449	*MAT****a*** *ura3Δ0 his3Δ0 leu2Δ0 sml1Δ0*::*KanMX URA3-Flag^5^-mec1-2369G-HIS3 rpn3-L140P*	This work

Growth comparisons were performed on YP media containing 2% glucose (YPD) or selective plates after 3–5 days at 30° unless stated otherwise. Standard concentrations used for the selections were 0.03% methyl methanesulfonate (Sigma-Aldrich), 6% ethanol, and 200 mM hydroxyurea (Sigma-Aldrich). Growth on plates containing MG-132 (Calbiochem) was adapted from Liu *et al.* (2007) using conditions that permeabilize cells. Strains were grown overnight in synthetic complete media lacking uracil and containing 0.1% proline as the nitrogen source, then 4 hr in the same media also containing 0.003% sodium dodecyl sulfate (SDS). Serial dilutions were spotted onto synthetic complete media containing 0.003% sodium dodecyl sulfate and 10, 25, or 50 μM MG-132 and grown at 30°.

### DNA molecules

An integrative vector to generate a Flag^5^ (pCB6192) fusion of Mec1 was constructed from pCB2143 (Genereaux *et al.* 2012) by replacing *TRA1* flanking sequences with *MEC1* as *Sph*I-*Hin*dIII and *Not*I-*Eco*RI fragments using oligonucleotides 6288-1/6288-1 and 6288-3/6288-4 (Table 2). A 1.1-kbp *Hin*dIII genomic fragment encoding *URA3* was inserted into this molecule to allow selection. The plasmid pCB6295 allowing integration of eGFP was created from pCB2143 by the replacement of the Flag cassette with a *Bam*HI-*Not*1 cassette encoding eGFP (Hoke *et al.* 2010). The *mec1* mutant alleles were constructed by polymerase chain reaction (PCR) using the oligonucleotides indicated in Table 2 and inserted into pCB2317 (*mec1-W2368A*). Myc^9^-tagged *LCD1* and *RPN3* were expressed from the *DED1* promoter in YCplac111, a *LEU2* centromeric plasmid, by inserting a *Not*I-*Sst*I fragment amplified from genomic DNA using oligonucleotides 6486-1/6486-1 and 6518-1/6522-1, respectively, downstream of the *DED1* promoter-myc^9^ cassette (Hoke *et al.* 2010). For integration in cells, *rpn3-L140P* including its native promoter was synthesized by PCR using oligonucleotides 6577-1 and 6510-2 and cloned as a *Hin*dIII-*Eco*R1 fragment into YIPlac211.

**Table 2:** Oligonucleotides used in this study

Number	Sequence	Description
6288-1	ATAAGGCGGCCGCCATGGAATCACACGTCAA-ATATCTTG	5′ coding region of *MEC1* to insert tags
6288-2	Atatgtcgaccgcctcataaaccatattctgtg	5′ coding region of *MEC1* to insert tags
6288-3	Ttcgcatgccttttcaaggctccataactat	*MEC1* promoter region to insert tags
6288-4	Ggaaagcttggagcgtgcgttccatcta	*MEC1* promoter region to insert tags
6313-1	AAGCTTGCATGCGTTGATGAATGTG	3′ coding region of *MEC1* for cloning of deletions
6313-2	Gcgtgatcaaaatggaagccaaccaatatac	*mec1-Δ1*
6338-1	Gcgtgatcatggaagccaaccaatatacatc	*mec1-Δ2*
6338-2	gcgtgatcaaccccaaaatggaagccaacc-aatatacatc	*mec1-2369G*
6349-3	Gcgtgatcaaagccaaccaatatacatcttgc	*mec1-Δ3*
6408-1	Cccagtccgccctgagcaaag	eGFP to confirm integration of tag
6408-2	Ccgtaaaattcgacacatgctttg	5′ coding of *MEC1* to confirm integration of tags
6486-1	ATAAGAATGCGGCCGCGATGAGACGAGAAACGGTGGG	5′ coding of *LCD1*
6486-2	CGGAATTCCAAACCGGTTCTGCTAAG	3′ coding of *LCD1*
6518-1	ATAAGAATGCGGCCGCAATGGCTAGCACTGCAGTAAT	5′ coding of *RPN3*
6522-1	Cggaattcgcgcccttataagaatcccaaatcg	3′ coding of *RPN3*
6577-1	CCCAAGCTTCGGAGTACGACCAGACGCTGA	Promoter of *RPN3*

### Fluorescence microscopy

Yeast cells expressing eGFP or RFP fusions were grown in synthetic complete media to stationary phase, then diluted 1:20 into synthetic complete media and grown for 6 hr with shaking. Fluorescent images were obtained using a Zeiss Axioskop 2 microscope driven by ImageJ 1.41 software (National Institutes of Health, Bethesda, MD) and a Scion CFW Monochrome CCD Firewire Camera (Scion Corporation, Frederick, MD) using RFP and GFP filter sets.

### Protein extracts and immunoprecipitation

Yeast strains were grown in YP media containing 2% glucose to an A_600_∼3.0. Extracts were prepared cryogenically as previously described (Saleh *et al.* 1997) . For immunoprecipitations, all steps were performed at 4°; 4.0 mg extract from the Mec1 derivative strains or 2 mg wild-type extract was suspended in immunoprecipitation (IP) buffer (50 mM HEPES, pH 7.5; 100 mM KCl; 0.1 mM EDTA; 0.2% Tween20; 1.0 mM dithiothreitol) containing protease inhibitors (1.0 mM phenylmethylsulfonyl fluoride, 1.0 mM benzamadine, 50 μg/ml trypsin inhibitor, 5 μg/ml pepstatin, and 5 μg/ml leupeptin; Sigma-Aldrich). Immunoprecipitations were performed with 100 μl 50% slurry of anti-Flag M2 magnetic beads (Sigma-Aldrich) and rotated for 2.5 hr. Beads were washed five times with 1.0 ml IP buffer. Protein was eluted in 1× SDS loading buffer (without reducing agent) at 65° for 6 min, then transferred to a fresh tube. Dithiothreitol was added to 20 mM and the sample was heated at 65° for 2 min.

### Protein kinase assays

Proteins captured by immunoprecipitation as described above were processed as in Mallory and Petes (2000) with modifications. Washed anti-Flag M2 beads (30 μl) containing the immunoprecipitated proteins were resuspended at a ratio of 1:1 in kinase buffer (10 mM HEPES-NaOH, pH 7.4; 50 mM NaCl; 10 mM MnCl_2_; 1 mM DTT). Kinase reactions contained the washed beads in which buffer was removed and 11.5 μl kinase buffer, 1.5 μl 200-μM ATP, 10 μCi γ-^32^P–labeled ATP (4500 Ci/mmol; 1 Ci = 37 GBq), and 1 μl rat 4E-BP1 (1 μg/μl; Santa Cruz Biotechnology). The reactions were performed at 30° for 30 min. Protein was eluted in 1× SDS loading buffer, boiled for 2 min, and then separated on a 15% Tris-Tricine PAGE gel. The gel was stained with Coomassie brilliant blue, destained, and incubated 30 min in 20% polyethylene glycol 400 and 50% methanol. The gel was dried, exposed to a storage phosphor screen, and analyzed on a Storm 860 Phosphorimager (GE Healthcare Life Sciences). Densitometry analysis of the gel image was performed using ImageQuant 5.2 (Molecular Dynamics).

### Gel filtration chromatography

Yeast extract (4.5 mg) prepared in 50 mM sodium phosphate (pH 7.0; 150 mM NaCl) was loaded at a flow rate of 0.3 ml/min onto a 24-ml FPLC Superose 6HR10/30 column (Amersham Pharmacia Biotech). Protein from 20-μl aliquots of 250-μl fractions was resolved by SDS-PAGE and proteins were detected by Western blotting. Densitometric scanning of films was performed using AlphaImager 3400 software (Alpha Innotech, San Leandro, CA).

### Western blotting

Western blotting was performed using polyvinylidene fluoride membranes and anti-Flag (M2; Sigma-Aldrich) or anti-Myc antibodies as described by Mutiu *et al.* (2007) and Hoke *et al.* (2010).

### Selection of suppressor strains

CY6106 (*mec1-W2368A-URA3*) was grown to stationary phase in YPD. In two separate experiments, 10 μl culture, approximately 2 million cells, was plated onto each of five YPD plates and ultraviolet-irradiated at a wavelength of 302 nm for 10 s. Survival was approximately 10%. Colonies growing at 37° were colony-purified under nonselective conditions and reanalyzed for growth at 37°. The suppressor strains were crossed with the *HIS3*-tagged *mec1-W2368A* strain, CY6077, to determine linkage of the suppressor with *mec1-W2368A*. In the two selections, nine strains had an unlinked suppressor mutation that segregated in a 2:2 fashion. Spore colonies of each were backcrossed with BY4741 or BY4742 six times and a nonmutated *mec1-W2368A* strain three times at each stage, selecting for temperature resistant spore colonies. The final isolates were sent for genomic sequencing as described. The *rpn3* mutation was verified after isolation of genomic DNA, PCR with oligonucleotides 6510-1 and 6510-2 (Table 2), and sequencing of the PCR product using oligonucleotide 6510-1 as primer.

### Genomic sequence analysis

Genomic DNAs were prepared from CY6076, CY6077, and four sister spore colonies from the final backcross of each of suppressor strains as described previously (Genereaux *et al.* 2012). The DNA from CY6076 and from CY6077 were pooled, as were those from each of the identical suppressor strains, and 5 µg DNA from each pooled sample was sent to the Biodiversity Research Centre (University of British Columbia, Vancouver, Canada) for DNA library construction and next-generation sequencing using 100-bp paired-end reads with the Illumina HiSequation 2000 platform. The *S. cerevisiae* genome sequence was downloaded from the *Saccharomyces* Genome Database (http://www.yeastgenome.org) on March 24, 2011. Custom bash and Perl scripts were written for the sequencing analysis. The program Bowtie (Langmead *et al.* 2009), allowing up to three mismatches per read, was used to map the reads to each chromosome of the yeast genome and output mapped reads in SAM format (Sequence Alignment/Map; Li *et al.* 2009). The variant call format from SAMtools (Li *et al.* 2009) was used to obtain a raw list of polymorphisms from the mapped reads. Those reads with a Phred quality score less than 20 were eliminated to obtain a filtered list of polymorphisms. A custom Perl script was written to identify those polymorphisms that were unique to the suppressor strain.

## Results

**Figure 1:**
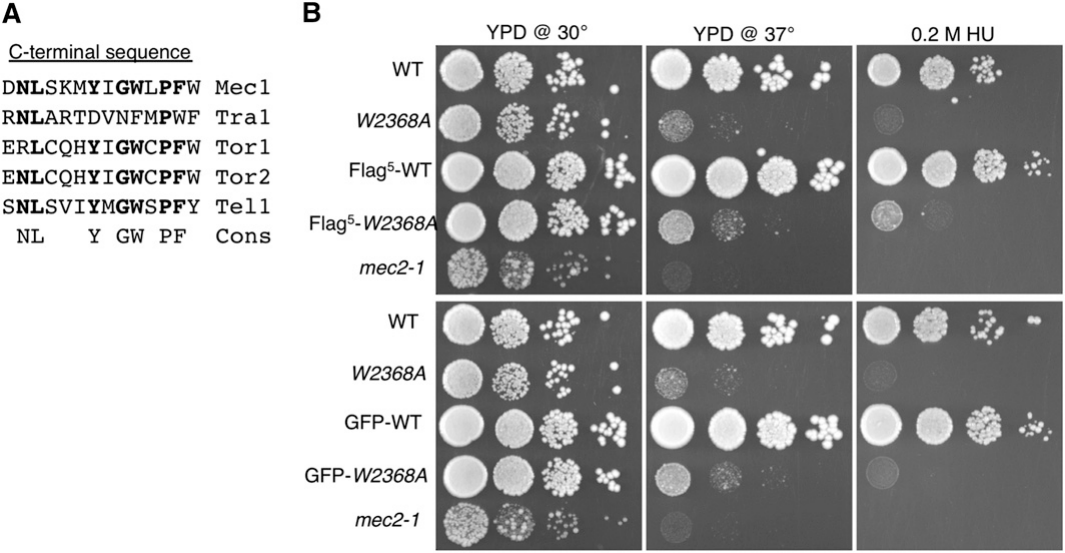
Phenotype of Flag^5^ and GFP-tagged *MEC1* strains. (A) Conservation of hydrophobic residues at the C-terminus of phosphoinositide three-kinase-related kinase (PIKK) family members. The C-terminal residues of the five PIKK family members found in *S. cerevisiae* are shown with the following consensus. (B) Yeast strains BY4741 [*MEC1*; wild-type (WT)], CY6076 (*mec1-W2368A*), CY6194 (Flag^5^-*MEC1*), CY6175 (Flag^5^-*mec1-W2368A*), a *mec2-1* strain (Weinert *et al.* 1994; included to verify the hydroxyurea plates), CY6307 (*GFP-MEC1*), and CY6302 (*GFP-mec1-W2368A*) were grown to stationary phase diluted 1/10^2^ and 10-fold serial dilutions were spotted onto selection plates as follows: YPD at 30°, YPD at 37°, and YPD at 30° containing 0.2 M hydroxyurea (HU).

The penultimate and terminal amino acid residues of the PIKK molecules are hydrophobic in family members (Figure 1A). Changing the terminal phenylalanine of Tra1 to alanine (*tra1*-F3744A) results in stress-related phenotypes and partial mislocalization of the protein to the cytoplasm (Genereaux *et al.* 2012). This mutation was suppressible by a F328S change in the chaperone component Tti2. A similar mutation in the terminal tryptophan of Mec1 (Mec1-W2368A) results in temperature-sensitive growth but is not suppressible by *tti2-F328S* (Genereaux *et al.* 2012). Here, we characterize Mec1 derivatives altered at their C-terminus to determine if the FATC domain has a general role in stability of PIKK family members. For these studies we constructed strains that express a genomically encoded allele with either five tandem copies of the Flag epitope (Flag^5^) or eGFP tag positioned at the N-terminus. As shown in Figure 1B, neither the Flag^5^ nor the eGFP tag alters growth of a strain with the otherwise wild-type allele in YPD media at 30°, 37°, or in the presence of 0.2 M hydroxyurea, a competitive inhibitor of ribonucleotide reductase (compare wild-type with Flag^5^–wild-type and GFP–wild-type). Growth of a strain with the *mec1-W2368A* allele also was examined. The *mec1-W2368A* strain was sensitive to hydroxyurea and, as shown previously (Genereaux *et al.* 2012), was temperature-sensitive. Neither Flag^5^ nor eGFP tags decreased the growth of the *mec1-W2368A* strain under these conditions.

**Figure 2:**
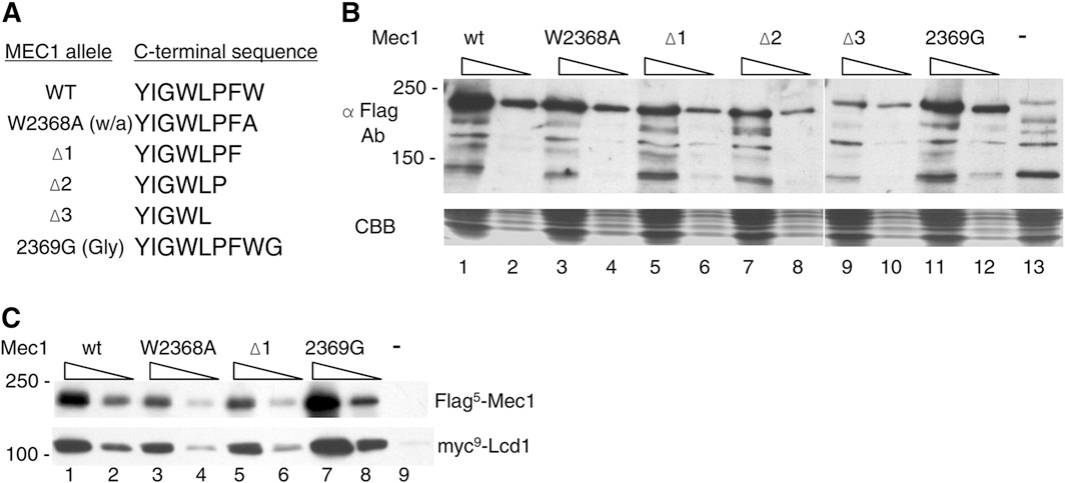
Expression of Mec1 derivatives with C-terminal mutations. (A) Amino acid sequences of the Mec1 derivatives. (B) Yeast strains CY6172 (*Flag^5^-MEC1*; lanes 1 and 2), CY6192 (*Flag^5^-mec1-W2368A*; lanes 3 and 4), CY6184 (*Flag^5^-mec1-Δ1*; lanes 5 and 6), CY6250 (*Flag^5^-mec1-Δ2*; lanes 7 and 8), CY6233 (*Flag^5^-mec1-Δ3*; lanes 9 and 10), CY6203 (*Flag^5^-mec1-2369G*; lanes 11 and 12), and BY4743 (lane 13) were grown in YPD media to mid-log phase and extracts were prepared by grinding in liquid nitrogen. Extracts were solubilized in the presence of protease inhibitors (1.0 mM phenylmethylsulfonyl fluoride, 5 μg/ml pepstatin, 1.0 mM benzamadine, 50 μg/ml trypsin inhibitor, and 5 μg/ml leupeptin); 50 μg and 10 μg protein were separated by sodium dodecyl sulfate (SDS)-PAGE; 50 μg was used for BY4743. The top portion of the gel was Western-blotted with anti-Flag (M2) antibody; the bottom portion was stained with Coomassie brilliant blue. (C) Interaction with Lcd1/Ddc2; 3 mg protein extract from yeast strains CY6172 (*Flag^5^-MEC1*; lanes 1 and 2), CY6192 (*Flag^5^-mec1-W2368A*; lanes 3 and 4), CY6184 (*Flag^5^-mec1-Δ1*; lanes 5 and 6), CY6203 (*Flag^5^-mec1-2369G*; lanes 7 and 8), and BY4743 (lane 9) containing YCplac111-myc^9^-Lcd1 were immunoprecipitated with anti-Flag antibody; 20 μl (odd-numbered lanes) and 5 μl of the immunoprecipitates were separated by SDS-PAGE and Western-blotted with anti-Flag antibody (upper panel) or anti-myc antibody (lower panel).

Four additional *mec1* alleles were constructed and integrated into a diploid strain. *mec1*-Δ1, *mec1*-Δ2, and *mec1*-Δ3 contain deletions of one, two, or three residues at the C-terminus of Mec1 (Figure 2A). Three deletions were constructed to decrease the likelihood that the C-terminal carboxyl might be artificially repositioned in a hydrophobic pocket in all of the derivatives. Mec1-2369G contains an additional glycine residue at the C-terminus. A comparable addition to Tra1 results in inviability (Hoke *et al.* 2010). After sporulation, there was a 2:2 segregation of viable and inviable spore colonies for each, indicating that insertion or deletions to the C-terminus of Mec1 compromise viability. To determine if the C-terminal mutations affect expression of Mec1, diploid strains containing a single copy of Flag^5^-tagged wild-type or mutant Mec1, and an untagged wild-type allele were analyzed by Western blotting. As shown in Figure 2B, all of the *mec1* alleles were expressed when cells were grown at 30°. Flag^5^-Mec1-W2368A and Flag^5^-Mec1-2369G were found at nearly wild-type levels. The deletion proteins were somewhat reduced, with the reduction paralleling the extent of the deletion. We also examined if the different *mec1* alleles interacted with Lcd1/Ddc2 (ATRIP in mammalian cells), an association required for the checkpoint functions of Mec1 (Rouse and Jackson 2000; Paciotti *et al.* 2000; Wakayama *et al.* 2001). Immunoprecipitations were performed with anti-Flag antibody and extracts prepared from strains containing myc^9^-tagged Lcd1/Ddc2 and a Flag^5^-tagged Mec1 derivative. As shown in Figure 2C, the ratio of myc^9^-tagged Lcd1/Ddc2 to Flag^5^-Mec1 was similar for each of the derivatives, indicating that Lcd1/Ddc2 interacted with Mec1-W2368A, Mec1-Δ1, and Mec1-G2369 as efficiently as the wild-type protein.

### Terminal residues of Mec1 are required for full kinase activity

**Figure 3:**
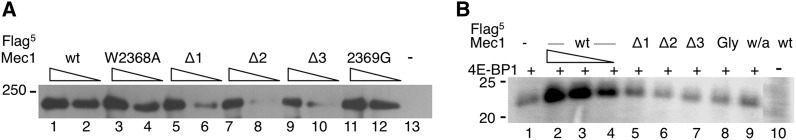
The terminal residues of Mec1 are required for its kinase activity. (A) Protein extracts were prepared from yeast strains CY6172 (*Flag^5^-MEC1*; lanes 1 and 2), CY6192 (*Flag^5^-mec1-W2368A*; lanes 3 and 4), CY6184 (*Flag^5^-mec1-Δ1*; lanes 5 and 6), CY6250 (*Flag^5^-mec1-Δ2*; lanes 7 and 8), CY6233 (*Flag^5^-mec1-Δ3*; lanes 9 and 10), CY6203 (*Flag^5^-mec1-2369G*; lanes 11 and 12), and BY4743 (lane 13) in the presence of protease inhibitors; 2 mg protein from CY6192 and 4 mg protein from the other strains were immunoprecipitated. One-third of the beads was suspended in 100 μl of 1× sodium dodecyl sulfate (SDS) loading buffer, boiled, and 10 μl or 5 μl were separated by SDS-PAGE (5% gel) and Western blotted with anti-Flag antibody. The BY4743 sample contained 10 μl bead suspension. (B) Phosphorylation assays. Two-thirds of the immunoprecipitate in (A) was suspended in 60 μl kinase buffer. Aliquots [8, 4, and 2 μl for wild-type (WT), 8 μl of the other samples] were used in kinase assays at 30° with 4E-BP1 as substrate (with the exception of lane 10 which contained buffered glycerol). Lane 1, BY4743; lanes 2–4 and 10, CY6172 (*Flag^5^-MEC1*); lane 5, CY6184 (*Flag^5^-mec1-Δ1*); lane 6, CY6250 (*Flag^5^-mec1-Δ2*); lane 7, CY6233 (*Flag^5^-mec1-Δ3*); lane 8, CY6203 (*Flag^5^-mec1-2369G*); and lane 9, CY6192 (*Flag^5^-mec1-W2368A*). Reactions were stopped with 3× SDS loading buffer and protein was separated on a 15% Tris-Tricine gel. The gel was fixed and processed as described in Materials and Methods. Lane 10 was analyzed on the same gel but was moved proximal to the other samples.

Because the phenotypes of the Mec1 derivatives were not attributable to their lack of expression or reduced interaction with Lcd1, we performed *in vitro* kinase assays to determine if this activity was impaired. We assayed the activity of Flag^5^-tagged Mec1, Mec1-Δ1, Mec1-Δ2, Mec1-Δ3, Mec1-2369G, and Mec1-W2368A after isolating the proteins by immunoprecipitation on Flag-antibody resin from diploid yeast strains. The immunoprecipitates of the Flag^5^-tagged proteins used in the kinase assays are shown in Figure 3A. Kinase assays were performed with rat 4E-BP1 (PHAS-1) as the substrate (Lin *et al.* 1994; von Manteuffel *et al.* 1996). The results for an assay performed at 30° are shown in Figure 3B. Immunoprecipitates from a strain wild-type for *MEC1*, but without tagged protein (BY4743; lane 1), and from CY6172 (Flag^5^-tagged Mec1; lane 10) in the absence 4E-BP1 were performed to control for nonspecific phosphorylation and to identify 4E-BP1, respectively. Phosphorylation of 4E-BP1 was 7.5-fold greater than background with the wild-type protein (compare lanes 1 and 2). Interestingly, phosphorylation by Flag^5^-Mec1-W2368A was less than 10% of that found for the wild-type protein (compare lanes 2 and 9). Although 4E-BP1 is not necessarily indicative of all native Mec1 substrates, this result suggests that only low levels of kinase activity are essential for viability in rich media. Phosphorylation of 4E-BP1 was not above background levels for Mec1-2369G (lane 8), likely explaining the inviability of the strain containing this derivative. Similarly, the kinase activity of the Mec1 deletion derivatives (lanes 5–7) was not above background, although exact comparison with the wild-type protein was difficult because of their somewhat reduced abundance in the immunoprecipitates.

### The terminal tryptophan residue is required for stability and localization of the protein

**Figure 4:**
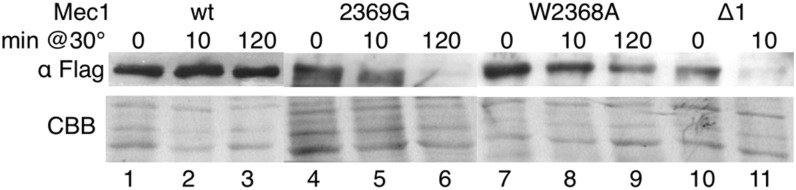
The terminal residues of Mec1 are required for its stability in extracts. Yeast strains CY6172 (*Flag^5^-MEC1*; lanes 1–3), CY6203 (*Flag^5^-mec1-2369G*; lanes 4–6), CY6192 (*Flag^5^-mec1-W2368A*; lanes 7–9), and CY6184 (*Flag^5^-mec1-Δ1*; lanes 10 and 11) were grown in YPD media to mid-log phase and extracts were prepared by grinding with glass beads in the absence of protease inhibitors. Extracts were then incubated at 30° for the indicated time period and 50 μg protein from each extract was separated by sodium dodecyl sulfate (SDS)-PAGE and Western-blotted with anti-Flag antibody. The lower panel is the lower portion of the gel after staining with Coomassie brilliant blue (CBB).

Based on the properties of Tra1 derivatives with C-terminal mutations (Genereaux *et al.* 2012), we predicted that the lack of kinase activity found for the Mec1 derivatives might result from improper folding of the molecules. The Mec1-W2368A, 2369G, and Δ1 derivatives were found within cells at levels approaching that of the wild-type when extracts were prepared with protease inhibitors and analyzed directly after isolation (Figure 2B). Predicting that the C-terminus was required for stability of the protein, we addressed whether the Mec1 derivatives would be susceptible to proteolysis in the absence of protease inhibitors. Protein was isolated from diploid strains containing Flag^5^ derivatives of Mec1, Mec1-2369G, Mec1-W2368A, and Mec1-Δ1. The extract was then incubated at 30° for 10 min or 2 hr, and aliquots were analyzed by Western blotting (Figure 4). The wild-type protein was stable in extracts with no obvious degradation in 2 hr. In contrast, each of the Mec1 derivatives showed some sign of degradation at the 10-min time point.

**Figure 5:**
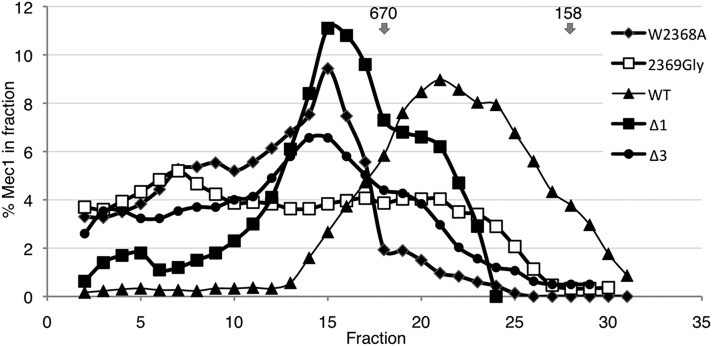
Resolution of Mec1 derivatives by size-exclusion chromatography. Protein extracts were prepared from yeast strains CY6172 (*Flag^5^-MEC1*), CY6192 (*Flag^5^-mec1-W2368A*), CY6184 (*Flag^5^-mec1-Δ1*), CY6233 (*Flag^5^-mec1-Δ3*), and CY6203 (*Flag^5^-mec1-2369G*); 4.5 mg protein from each strain was separated independently on a 24-ml FPLC Superose 6HR10/30 column (Amersham Pharmacia Biotech). Protein from 20 μl aliquots of 250 μl fractions was resolved by sodium dodecyl sulfate (SDS)-PAGE and proteins were detected by Western blotting. The amount of protein in each fraction was determined by densitometry and calculated as a ratio of the total protein. The plot shows the average of a three-fraction window. The arrows indicate the migration of 670 kD and 158 kD molecular mass standards.

As another measure of whether the Mec1 derivatives were present in their functional forms, we performed size exclusion chromatography on extracts prepared from strains containing Flag^5^-tagged versions. As shown in Figure 5, wild-type Mec1 elutes from a Superose 6 column with an estimated mass less than ∼670 kD. A portion of each of the mutant versions eluted from the column in a fraction with a significantly greater estimated mass, with none of the profiles paralleling the wild-type. Mec1-2369G had a distinct profile in that it was dispersed almost equally across the high-molecular-mass fractions. The presence of the higher-mass forms suggests the possibility of improper folding of the Mec1 derivatives, or perhaps prolonged association with chaperone complexes that are required for the formation of functional PIKK proteins (Boulon *et al.* 2012; Makhnevych and Houry 2012).

**Figure 6:**
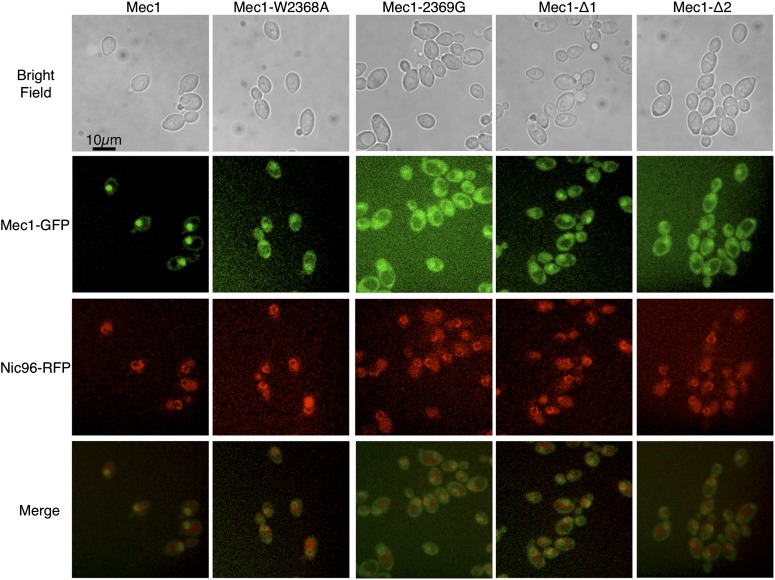
Localization of eGFP-Mec1. Diploid yeast strains containing an eGFP-tagged Mec1 derivative (CY6307, Mec1; CY6302, Mec1-W2368A; CY6330, Mec1-Δ1; CY6344, Mec1-Δ2; and CY6342, Mec1-2369G) were mated to a strain containing RFP-tagged Nic96 (Huh *et al.* 2003). Diploid strains were grown at 30° in synthetic complete (SC) media to stationary phase, diluted 1:20 in SC, grown another 6 hr, and visualized by fluorescence microscopy. A representative field of cells is shown.

We also examined the localization of N-terminally eGFP-tagged versions of the proteins. Diploid strains expressing wild-type Mec1 and a single copy of eGFP-tagged Mec1 or a derivative were grown at 30°. RFP-tagged Nic96 was used to mark the nuclear periphery. As shown in Figure 6, eGFP-tagged wild-type Mec1 is found principally in the nucleus. The distributions of each of Mec1-W2368A, Mec1-2369G, Mec1-Δ1, and Mec1-Δ2 are more disperse, with both nuclear and cytoplasmic localization apparent. The experiment also was performed for cells grown at 37°, but the signal was significantly reduced for the Mec1 derivatives.

### *rpn3-L140P* suppresses the temperature-sensitive growth of *mec1-W2368A*

**Figure 7:**
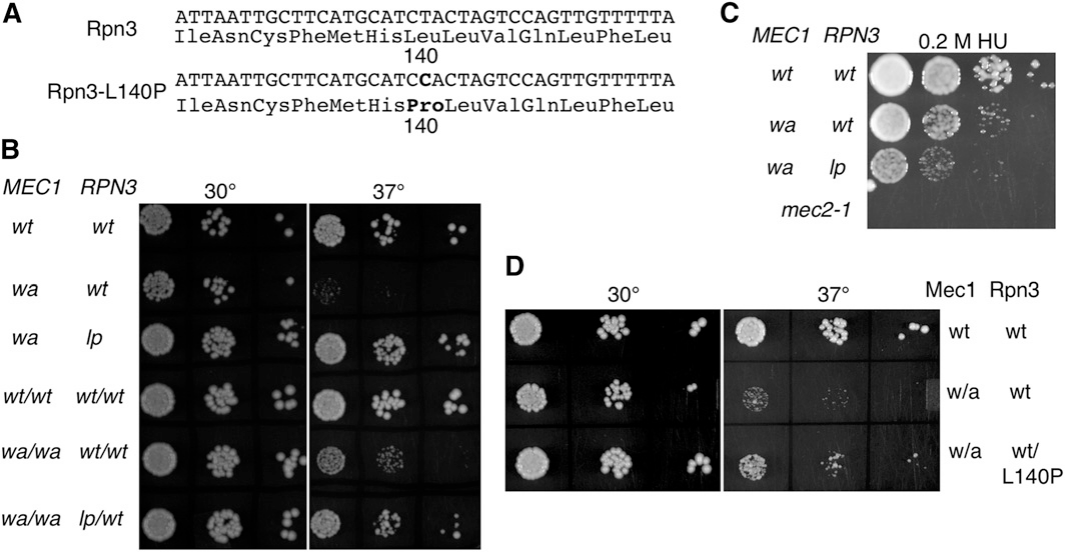
Suppression of the temperature-sensitive growth resulting from *mec1-W2368A* by *rpn3-L140P*. (A) Sequence of *RPN3* and the *rpn3-L140P* allele. (B) Yeast strains BY4742 (*MEC1 RPN3*), CY6077 (*mec1-W2368A RPN3*), CY6265 (*mec1-W2368A rpn3-L140P*), BY4743 (*MEC1/MEC1 RPN3/RPN3*), CY6368 (*mec1-W2368A/mec1-W2368A*), and CY6385 (*mec1-W2368A/mec1-W2368A RPN3/rpn3-L140P*) were grown to stationary phase, diluted 1/10^2^, and 10-fold serial dilutions were spotted onto YPD plates at 30° and 37°. (C) Serial dilutions of BY4742, CY6077, and CY6265 were spotted onto a YPD plate containing 0.2 M hydroxyurea. A *mec2-1* strain was included to verify the quality of the hydroxyurea. (D) Serial dilutions of BY4741, CY6076 (*mec1-W2368A RPN3*), and CY6465 (*mec1-W2368A RPN3/rpn3-L140P-URA3*) were spotted onto YPD plates at 30° and 37°.

Together these results suggested that the C-terminal residues of Mec1 were required for the folding/stability of the molecule. If this is the case, then we predict that it should be possible to identify second site suppressor mutations that restore the levels of the protein and allowed growth of the *mec1-W2368A* strain at 37°. We plated approximately 20 million cells at a density of 1 million cells per plate, subjected them to a low dose of ultraviolet radiation, and selected colonies that grew at 37°. Nine colonies were identified that carried a single extragenic suppressor mutation. These were backcrossed an additional nine times to eliminate variations unlinked to the suppressor mutation, and then their genomic sequence was determined. Unique mutations were identified in the following four strains: *rpn3* (L140P; a T-to-C transition at bp 419; Figure 7A); *rfx1* (S213N); *rfx1* (S462L); and *sml1* (L62ochre). Loss of function of Sml1 and loss of function of Rfx1 are known to suppress nonfunctional alleles of *mec1* (Weinert *et al.* 1994; Desany *et al.* 1998; Huang *et al.* 1998). Consistent with these being causative mutations, we found that our *sml1* and *rfx1* strains also would suppress *mec1-Δ1*. In contrast, the *rpn3-L140P* allele would not suppress *mec1-Δ1*, *mec1-Δ2*, or *mec1-2369G*.

Because Rpn3 encodes a component of the 19S proteasomal regulatory particle (Kominami *et al.* 1997), thus potentially linking it to the stability of Mec1, we chose to examine *rpn3-L140P* in greater detail. The strain containing this mutation grew at 37°, at a level comparable with a wild-type strain, and the mutation responsible did so in a partially dominant fashion (Figure 7B). The suppressor mutation did not facilitate growth on plates containing 0.2 M hydroxyurea, but rather resulted in a further reduction of growth (Figure 7C). Two approaches were used to demonstrate that suppression of *mec1-W2368A* was the result of *rpn3-L140P*. First, we compared the partially dominant effect of *rpn3-L140P/RPN3* as shown in Figure 7B, with the ability of *rpn3-L140P* inserted into CY6076 (*mec1-W2368A*) on an integrating plasmid (Figure 7D). As was found for the heterozygous diploid, addition of the *rpn3-L140P*–containing plasmid partially, but not completely, reversed the slow growth at 37° because of *mec1-W2368A*. Second, we analyzed independent spore colonies from a cross of CY6265 (*rpn3-L140P mec1-W2368A*) and CY6076 (*RPN3mec1-W2368A*). The *RPN3* allele from each spore colony was isolated by PCR and sequenced. For 14 alleles (seven fast-growing strains and seven slow-growing strains), the fast growth predicted the presence of the *rpn3-L140P* allele.

**Figure 8:**
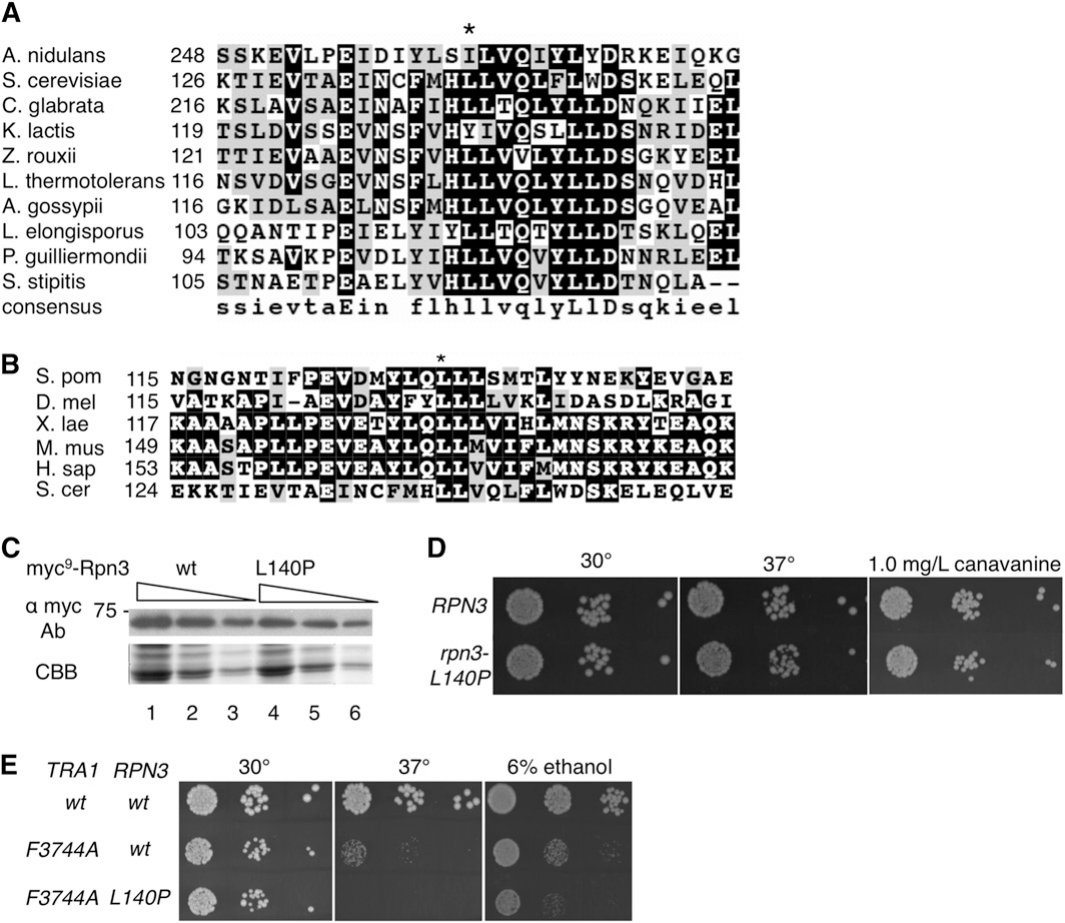
Leucine 140 is found in a conserved hydrophobic region. (A) Multiple sequence alignment of Rpn3 from a variety of fungal species. Leucine 140 (*S. cerevisiae*) is indicated by an asterisk. The alignment was performed with MUSCLE (Edgar 2004). In order, the proteins are as follows: *Aspergillus* nidulans, XP_660371.1; *Saccharomyces cerevisiae*, NP_010938.1; *Candida glabrata*, XP_447606.1; *Kluyveromyces lactis*, XP_453544.1; *Zygosaccharomyces rouxii*, XP_002496514.1; *Lachancea* thermotolerans, XP_002554976.1; *Ashbya gossypii*, NP_983112.1; *Lodderomyces elongisporus*, XP_001526739.1; *Pichia guilliermondii*, XP_001487379.1; and *Scheffersomyces stipitis*, XP_001383755.2. (B) Multiple sequence alignment of Rpn3 from a variety of eukaryotes. The proteins are as follows: *Schizosaccharomyces pombe*, NP_595282.2; *Drosophila melanogaster*, NP_477300; *Xenopus laevis*, NP_001085955.1; *Mus musculus*, NP_033465.1; and *Homo sapiens*, NP_002800.2. (C) Expression of Rpn3-L140P. Extracts were prepared from BY4741 expressing myc^9^-Rpn3 (lanes 1-3) or myc^9^-Rpn3-L140P from a centromeric plasmid; 50, 25, and 10 μg of protein was separated by sodium dodecyl sulfate (SDS)-PAGE and Western-blotted with anti-Myc antibody (upper panel). The lower section of the gel was stained with Coomassie brilliant blue (lower panel). (D) Phenotype of *rpn3-L140P* in isolation. Yeast strains BY4741 (*RPN3*) and CY6398 (*rpn3-L140P*) were grown to stationary phase and serial dilutions were spotted onto YPD plates at 30° and 37° and onto a minimal plate containing 1.0 mg/l canavanine. (E) *rpn3-L140P* does not suppress *tra1-F3744A*. Yeast strains CY4353 (*TRA1 RPN3*), CY4350 (*tra1-F3744A RPN3*), and CY6418 (*tra1-F3744A rpn3-L140P*) were grown to stationary phase and serial dilutions were spotted onto YPD plates at 30° and 37° or a YPD plate containing 6% ethanol at 30°.

Rpn3 is an essential 524-amino-acid residue protein that contains PCI/PINT-associated module (PAM) and winged helix domains located C-terminally distal to residue 140. As shown in Figure 8, A and B, L140 is in a hydrophobic region conserved in fungal species and more broadly in eukaryotes. The L140P mutation does not alter the level of Rpn3 found in the cell (Figure 8C). To determine the effect of Rpn3-L140P in isolation, yeast strain CY6398 (*MEC1rpn3-L140P*) was engineered by backcrossing CY6265 with BY4741. As shown in Figure 8D, the *rpn3-L140P* allele had no detectable effect on growth of cells in rich media at 30°. A slight reduction in growth was observed for cells grown at 37° and in media containing the arginine analog canavanine.

As mentioned, *rpn3-L140P* does not suppress *mec1-Δ1*, *mec1-Δ2*, or *mec1-2369G*. To address whether *rpn3-L140P* would suppress a mutation similar to *mec1-W2368A* in a related PIKK protein, we introduced *rpn3-L140P* into a strain containing a Phe-to-Ala change of the terminal phenylalanine of Tra1 (*tra1-F3744A*). As shown in Figure 8E, *rpn3-L140P* did not suppress the slow growth at 37° or in ethanol-containing media caused by *tra1-F3744A*, and, in fact, resulted in synthetic slow growth in this context.

**Figure 9:**
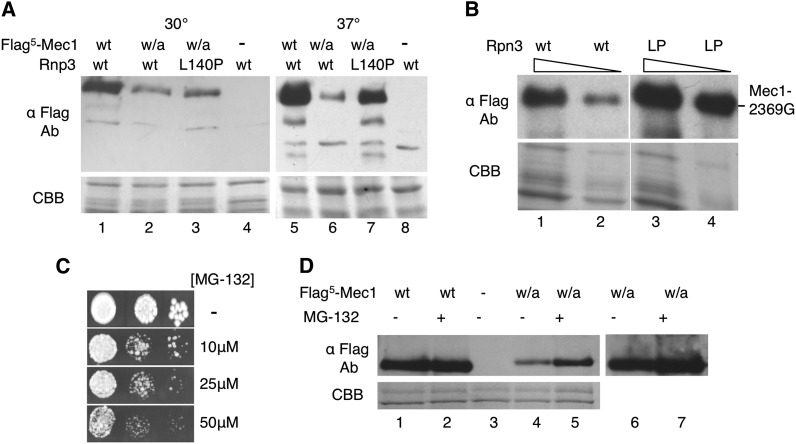
*rpn3-L140P* increases the level of C-terminally altered Mec1 derivatives. (A) Mec1-W2368A. Protein extracts were prepared by bead lysis in buffer lacking protease inhibitors from diploid yeast strains CY6172 (*MEC1/Flag^5^-MEC1 RPN3/RPN3*), CY6192 (*MEC1/Flag^5^-mec1-W2368A RPN3/RPN3*), CY6400 (*MEC1/Flag^5^-mec1-W2368A rpn3-L140P/rpn3-L140P*), and BY4743 (*MEC1/MEC1 RPN3/RPN3*) grown at 30° or 37°; 50 μg of protein was separated by sodium dodecyl sulfate (SDS)-PAGE and the upper portion of the gel was Western-blotted with anti-Flag antibody. The lower part of the gel was stained with Coomassie brilliant blue (CBB). (B) Mec1-2369G. Yeast strains CY6349 (*mec1-2369G RPN3 sml1Δ0*::*KanMX*; lanes 1 and 2) and CY6449 (*mec1-2369G rpn3-L140P sml1Δ0*::*KanMX*; lanes 3 and 4) were grown in YPD media for 8 hr at 37°; 40 μg (odd lanes) and 20 μg (even lanes) of protein extract was separated by SDS-PAGE and Western-blotted with anti-Flag antibody or stained with CBB. (C) BY4742 was grown in media containing proline as the nitrogen source and 0.003% SDS for 3 hr, then serial dilutions were plated onto identical synthetic complete media with the indicated amount of MG-132. (D) CY6172 (*MEC1/Flag^5^-MEC1*; lanes 1 and 2), BY4743 (lane 3), and CY6192 (*MEC1/Flag^5^-mec1-W2368A*; lanes 4–7) were grown in media containing proline as the nitrogen source and 0.003% SDS for 3 hr at 37°. MG-132 was added to a final concentration of 75 μM (+) or the equivalent volume of dimethylsulfoxide (−) and the cells were grown for an additional 2 hr. Extracts were prepared by glass bead lysis and 30 μg was separated by SDS-PAGE. The upper portion of the gel was Western-blotted with anti-Flag antibody. The lower portion was stained with CBB. Lanes 6 and 7 are an overexposure of lanes 4 and 5.

The terminal tryptophan-to-alanine mutation in Mec1 reduced the stability of the protein (Figure 4). To investigate the effect of *rpn3-L140P* on Mec1-W2368A, the diploid strain CY6400 with the genotype *Flag^5^-mec1-W2368A/MEC1rpn3-L140P/rpn3-L140P* was engineered. The level of Flag^5^-Mec1 in this strain was compared to that found in a wild-type *RPN3/RPN3* background (Figure 9A). For extracts prepared from cells grown at 30° and in the absence of protease inhibitors, the level of Mec1-W2368A was somewhat reduced relative to the wild-type (Figure 9A, compare lanes 1 and 2). The *rpn3-L140P* resulted in an increase in Mec1-W2368A (Figure 9A, compare lanes 2 and 3). For extracts prepared from cells grown at 37°, the level of Mec1-W2368A was further reduced relative to the wild-type (Figure 9A, compare lanes 5 and 6), with *rpn3-L140P* resulting in a dramatic increase (Figure 9A, compare lanes 6 and 7). We conclude that *rpn3-L140P* increases the level of Mec1-W2368A, thus facilitating growth at elevated temperatures. As shown in Figure 9B, *rpn3-L140P* acts more broadly, increasing the level of Mec1-2369G when cells are grown at 37°.

The suppression of *mec1-W2368A* by *rpn3-L140P* introduces the possibility that Mec1 turnover is mediated by its degradation by the proteasome. To begin to address this issue, we analyzed Flag^5^-tagged Mec1 and Mec1-W2368A in cells treated with the proteasome inhibitor MG-132. Cells were treated under conditions described by Liu *et al.* (2007) to increase their permeability to MG-132. As shown in Figure 9C, when grown in media containing proline as the nitrogen source and 0.003% SDS, yeast is sensitive to MG-132 in micromolar concentrations. For the analysis of Mec1 and Mec1-W2368A, extracts were prepared from cells grown at 37° in the presence or absence of 75 μM MG-132 for 2 hr. Wild-type Mec1 was not altered by the MG-132 treatment (Figure 9D, compare lanes 1 and 2). In contrast, Mec1-W2368A levels increased approximately two-fold on treatment (Figure 9D, compare lanes 4 and 5), suggesting that the proteasome contributes, at least in part, to the turnover of the protein. For the wild-type and Mec1-W2368A proteins, there was no evidence of higher-molecular-mass forms that would be indicative of ubiquitylation of the protein.

## Discussion

The FATC domain is found at the C-terminus of all the PIKK proteins. In each, the two most C-terminal residues are large and hydrophobic. We have shown that the terminal tryptophan residue of Mec1 is required for the function of the protein. Deletion of this residue results in a loss of viability; conversion to alanine results in temperature-sensitive growth and reduced growth in hydroxyurea, a condition in which deoxyribonucleotides are depleted. Moreover, the position of the terminal tryptophan is essential. Addition of a single glycine residue also causes loss of viability. Because these results parallel those found for Tra1 (Genereaux *et al.* 2012), we conclude that the terminal residues are likely a key feature of all the PIKK proteins, a conclusion consistent with the positioning of the FATC domain in the structure of mTOR (Yang *et al.* 2013).

We propose that at least one role for the terminal residue is assisting in the folding of the protein. The C-terminally deleted Mec1 proteins were less abundant in crude protein lysates, and all of the derivatives analyzed were unstable in cell extracts prepared in the absence of protease inhibitors. This suggests that the temperature-sensitive phenotype of the *mec1-W2368A* strain may result from decreased stability of the protein at elevated temperatures, a conclusion supported by the increased levels found in the *rpn3-L140P* strain or in the presence of MG-132. Also suggesting the possibility of misfolding, we found that whereas wild-type Mec1 was found almost exclusively in the nucleus, the partially active and inactive forms of the protein showed partial cytoplasmic localization. In addition, each of the mutant forms of Mec1 chromatographed aberrantly on a size-exclusion column. Structural models (Lempiäinen and Halazonetis 2009; Sturgill and Hall 2009; Yang *et al.* 2013) position the FATC domain in a hydrophobic pocket that in porcine PI3K-γ interacts with helical domains (Walker *et al.* 1999). Loss of hydrophobic interactions may decrease the stability of the PI3K domain, whereas addition of a glycine residue may not permit packing into the pocket. In both cases, the FATC domain and the core PI3K domain would be susceptible to proteolysis.

Our results with Mec1 closely parallel what we have observed with Tra1 (Hoke *et al.* 2010). In the case of Tra1, converting the terminal phenylalanine to alanine results in mislocalization, decreased protein levels, and a number of stress-related phenotypes. Consistent with a role for the FATC domain in folding, alleles of *tti2*, whose product with Tel2 and Tti1 is proposed to act as a chaperone (Horejsi *et al.* 2010; Hurov *et al.* 2010; Kaizuka *et al.* 2010; Takai *et al.* 2010), suppress *tra1-F3744A* in a partially dominant fashion (Genereaux *et al.* 2012). It was interesting that both Tra1-F3744A and Mec1-W2368A are mislocalized. Whether the other properties of the Mec1 and Tra1 derivatives result from or cause the mislocalization will require additional experimentation; however, our working model is that the molecules need to be specifically localized in the cytoplasm in complex with chaperones until they achieve a conformation that will support nuclear import and, if diverted from this pathway, are at risk of being degraded.

Mutation of the terminal residue of Mec1 to alanine diminished kinase activity to a level approximately 10-fold less than that of the wild-type protein. The decrease in kinase activity of Mec1-W2368A and the other Mec1 derivatives can be explained by a role for the terminal residues in folding and their possible participation in substrate recognition as found for mTOR (Yang *et al.* 2013). Because *mec1-W2368A* will support viability, minimal kinase activity must be sufficient for growth under optimal conditions. In media containing hydroxyurea, where replication fork collapse will increase, this same level of activity was insufficient. It is also this requirement for maximal levels of Mec1 activity during replicative stress that likely explains why *rpn3-L140P* would not suppress slow growth of the *mec1-W2368A* strain in hydroxyurea. We note that interpretation of this experiment is somewhat complicated because of the role of Rpn3 in aspects of cell-cycle control (Bailly and Reed 1999).

Lcd1/Ddc2 interacts with the N-terminal HEAT domain sequences of Mec1 (Wakayama *et al.* 2001), thus providing a rationale for why it interacted with each of the altered Mec1 proteins with approximately the same efficiency. Because some of these derivatives are partially excluded from the nucleus, this result suggests that the Mec1-Lcd1/Ddc2 interaction takes place in the cytoplasm.

We have shown that a mutation resulting in a change of leucine 140 to proline in Rpn3 suppresses the temperature-sensitive growth of a *mec1-W2368A* strain. Rpn3 is a component of the 19S regulatory particle of the 26S proteasome (Beck *et al.* 2012; Lander *et al.* 2012; Lasker *et al.* 2012; Kish-Trier and Hill 2013). The regulatory particle can be subdivided into a base and lid. The base, composed of 10 components, interacts directly with the 20S proteolytic core particle and contains ATPases (Rpt1-6) required for the unfolding of substrate proteins (Smith *et al.* 2007; Rabl *et al.* 2008; Tomko *et al.* 2010). In addition to Rpn3, the lid contains Rpn5–Rpn9, Rpn11, Rpn12, and Sem1. The 26S proteasome is the key enzymatic machinery required to degrade ubiquitylated proteins, either misfolded or short-lived, in the cell (Finley 2009). Although the exact role of Rpn3 is unknown, the lid is involved in recognizing ubiquitylated target proteins (van Nocker *et al.* 1996; Husnjak *et al.* 2008; Schreiner *et al.* 2008) and removing the ubiquitin chains before the degradation of the protein by the 20S proteolytic core particle (Verma *et al.* 2002). Also of note, independent functions for the 19S regulatory particle have been observed in nucleotide excision repair (Russell *et al.* 1999; Gillette *et al.* 2001) and transcription (Ferdous *et al.* 2001; Gonzalez *et al.* 2002; Lee *et al.* 2005; Uprety *et al.* 2012).

We have considered possible models for how *rpn3-L140P* may suppress *mec1-W2368A*. It is unlikely that Rpn3-L140P acts indirectly by altering the DNA damage response, as is seen with loss of function of Sml1 or Rfx1, because of the observed allele specificity and the increased levels of Mec1-W2368A in the *rpn3-L140P* background. Models for how *rpn3-L140P* suppresses the temperature sensitivity of *mec1-W238A* need to reflect the role of the terminal sequences in the folding, localization, or stability of the protein. We present two models that are not mutually exclusive. Key in these is that *rpn3-L140P* increases the level of Mec1-W2368A, and does so more for Mec1-W2368A than for wild-type Mec1, and also more at 37° than 30°. First, the proteasomal regulatory particle has chaperone activities that normally participate in the delivery of the protein target to the 20S core particle (Braun *et al.* 1999). Independently, this activity could aid in the folding of Mec1-W2368A at elevated temperature and could be enhanced by Rpn3-L140P. This model would account for the partial dominance of the *rpn3-L140P* allele and would be analogous to the suppression of *tra1-F3744A* by alleles encoding the chaperone component Tti2. Interestingly, the same *tti2* alleles do not suppress *mec1-W2368A* (Genereaux *et al.* 2012), and loss of interactions with the Tel2-Tti1-Tti2 component Tel2 only modestly affects Mec1 function (Anderson and Blackburn 2008), leaving the possibility that another chaperone may replace or augment their activity. In the second model, the proteasome is involved in the turnover of improperly folded or mislocalized Mec1-W2368A. Subtle changes in targeting or activity of the proteasome caused by the L140P substitution in Rpn3 could decrease the rate of turnover, affording additional time for Mec1-W2368A to fold into a stable conformation or to be correctly localized. The *rpn3* allele would have partial dominance by altering the activity of half of the proteasome particles. We favor this model because inhibiting the proteolytic activity of the proteasome with MG-132 also increased the level of Mec1-W2368A. In addition, the L140P mutation is in a highly conserved region of the protein, suggesting a functional role. Although the phenotypes attributable to *rpn3-L140P* were not severe under most conditions, it does result in synthetic slow growth in combination with *tra1-F3744A* and reduced growth in combination with *mec1-W2368A* in media containing hydroxyurea. We did not observe an obvious loss of mobility of Mec1-W2368A in any of our Western blots that would be indicative of Mec1 polyubiquitylation, the general signal for targeting to the proteasome. The model is actually simplified in the absence of Mec1-W2368A ubiquitylation because it is less likely that polyubiquitylated Mec1-W2368A could be restored to an active form. The question would remain, however, of how Mec1-W2368A might be targeted to the proteasome. Examples of proteasomal targeting not requiring direct ubiquitylation do exist, this targeting is mediated by a second molecule, which in some cases is ubiquitylated (Bercovich *et al.* 1989; Sheaff *et al.* 2000; Perrotti *et al.* 2000; Sdek *et al.* 2005; Isono *et al.* 2008). Because of the nature of the role of Rpn3 in the proteasome, we cannot exclude a third model whereby *rpn3-L140P* acts indirectly by altering the level or activity of an intermediary protein.

The *rpn3-L140P* mutation suppressed some (growth at 37°) but not all (growth in hydroxyurea) phenotypes resulting from *mec1-W2368A*. We believe that this can be attributed to the threshold of Mec1 activity required for growth in hydroxyurea being greater than that for growth at 37°. In turn, this suggests that Rpn3-L140P is not sufficient to increase the activity of Mec1-W2368A above this threshold. We have considered whether Rpn3-L140P is able to increase the kinase activity of Mec1-W2368A by analyzing the activity of Flag^5^-tagged Mec1-W2368A isolated by immunoprecipitation from wild-type *RPN3* and *rpn3-L140P* strains. The experiment was performed with cells grown at 30°, at which temperature the control *mec1-W2368A RPN3* strain is able to grow. After normalizing to the amount of Mec1-W2368A present, an increase in activity was not observed for the protein from the *rpn3-L140P* strain (Supporting Information, Figure S1). This result is consistent with *rpn3-L140P* suppressing *mec1-2368A* by increasing the level and localization of Mec1-W2368A protein rather than by increasing the per-molecule activity.

As indicated, the 19S regulatory particle has a role in transcriptional regulation. Rpn3-L140P thus could increase the level of active Mec1-W2368A by activating its transcription; however, several points argue against this. Of particular note, the suppression by *rpn3-L140P* was observed when Mec1-W2368A was expressed from both its native promoter, the case for the original selections, and from the *TRA1* promoter, the case for the Flag-tagged derivatives. Furthermore, a role in transcription would be expected to be manifest by increased protein levels of approximately the same magnitude at both 30° and 37°. Increased expression was greater for cells grown at 37°, at which temperature Mec1-W2368A would have greater tendency to misfold. The finding that MG-132 mimics *rpn-L140P* also supports a role for the mutation in altering protein turnover rather than transcription. Finally, unlike the ATPase encoding genes *RPT6* (*SUG1*) and *RPT4* (*SUG2*), alleles of *RPN3* were not identified in screens for altered transcriptional regulation (Swaffield *et al.* 1992; Xu *et al.* 1995; Russell *et al.* 1996).

In summary, we have shown that the C-terminus of Mec1 is important for the stability of the protein, a feature likely common to the PIKK family. The reduced kinase activity of Mec1-W2368A suggests the C-terminal residues participate in the activity of the protein, with the viability of the *mec1-W2368A* strain indicating that minimal kinase activity is required for growth in rich media. Furthermore, suppression of the temperature sensitivity of *mec1-W2368A* by an allele encoding the proteasome component Rpn3 implicates the proteasome in regulating Mec1 levels, and implicates Rpn3 as having a key role in this function.

## Supplementary Material

Supporting Information
