# Supplementary material for: The C-terminal Residues of Saccharomyces cerevisiae Mec1 Are Required for Its Localization, Stability, and Function
Source: G3 (Bethesda). 2013 Oct 1;3(10):1661–74. doi: 10.1534/g3.113.006841 (PMC3789791; doi:10.1534/g3.113.006841)
Supplement: Supporting Information [file supp_3_10_1661__index.html]

The C-terminal Residues of Saccharomyces cerevisiae Mec1 Are Required for Its Localization, Stability, and Function — Supporting Information 

# The C-terminal Residues of *Saccharomyces cerevisiae* Mec1 Are Required for Its Localization, Stability, and Function

## Supporting Information for DaSilva *et al.*, 2013

**Files in this Data Supplement:**

- Figure S1 - *rpn3-L140P* does not increase Mec1-W2368A kinase activity. (PDF, 559 KB)
