# Supplementary material for: The C-terminal Residues of Saccharomyces cerevisiae Mec1 Are Required for Its Localization, Stability, and Function
Source: G3 (Bethesda). 2013 Oct 1;3(10):1661–74. doi: 10.1534/g3.113.006841 (PMC3789791; doi:10.1534/g3.113.006841)
Supplement: Supporting Information [file supp_g3.113.006841_006841SI.pdf]

G3 · Genes | Genomes | Genetics

# The C-terminal Residues of *Saccharomyces cerevisiae* Mec1 are required for its Localization, Stability and Function

Lance F. DaSilva*, Samantha Pillon*, Julie Genereaux*, Megan J. Davey*, Gregory B. Gloor*, Jim Karagiannis§ and Christopher J. Brandl*

*Department of Biochemistry, Schulich School of Medicine & Dentistry, The University of Western Ontario, London Canada N6A5C1

§ Department of Biology, The University of Western Ontario, London Canada N6A 5B7

**DOI: 10.1534/g3.113.006841**

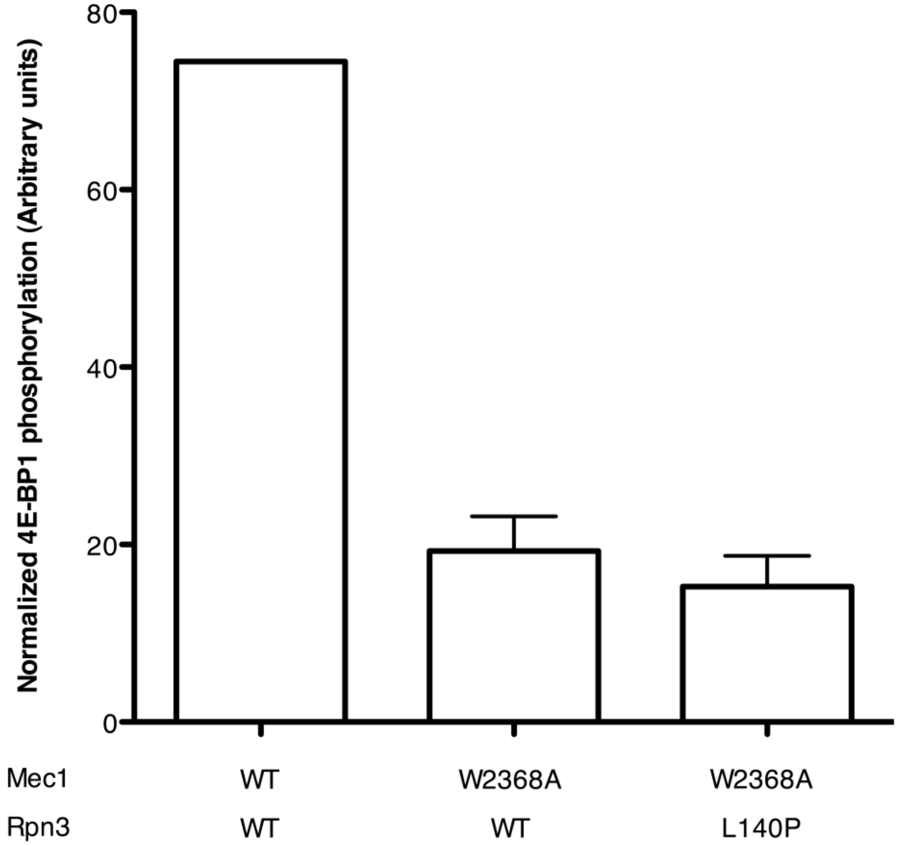


**Figure S1** *rpn3-L140P* does not increase Mec1-W2368A kinase activity. Protein extracts were prepared by bead lysis in buffer containing protease inhibitors from strains CY6194 (*Flag*$^{5}$*-MEC1 RPN3*), CY6175 (*Flag*$^{5}$*-mec1-W2368A RPN3*) and CY6391 (*Flag*$^{5}$*-mec1-W2368A rpn3-L140P*) grown at 30°. Two milligrams of protein was immunoprecipitated with anti-Flag M2 magnetic beads. Two-thirds of the immunoprecipitate was suspended in 60 µl of kinase buffer. Aliquots of four microliters were used in kinase assays performed at 30° with 4E-BP1 as substrate. Shown is the amount of 4E-BP1 phosphorylation normalized to Mec1 protein levels. Error bars represent the mean ± SD of three replicates.
